# Retraction: EEG signal connectivity for characterizing interictal activity in patients with mesial temporal lobe epilepsy

**DOI:** 10.3389/fneur.2023.1207981

**Published:** 2023-04-28

**Authors:** Frontiers Editorial Office

The journal retracts the 21 July 2021 article cited above.

Following publication, the authors contacted the Editorial Office to request the retraction of the cited article, stating that the code that was used for the analyses performed in the article contains an error. Therefore, the conclusions reported in the article are no longer supported by the analyses. An investigation was conducted in accordance with Frontiers' policies that confirmed this; and the article has been retracted.

This retraction was approved by the Chief Editor of Frontiers in Neurology and the Chief Executive Editor of Frontiers. The authors agree to this retraction.

